# Exercise as treatment for alcohol use disorder: A qualitative study

**DOI:** 10.1111/dar.13527

**Published:** 2022-09-08

**Authors:** Victoria Gunillasdotter, Sven Andréasson, Mats Hallgren, Maria Jirwe

**Affiliations:** ^1^ Department of Global Public Health Karolinska Institutet Stockholm Sweden; ^2^ Centre for Psychiatry Research Sweden Stockholm Health Services Stockholm Sweden; ^3^ Department of Health Sciences Swedish Red Cross University Huddinge Sweden; ^4^ Department of Neurobiology, Care Sciences and Society Karolinska Institutet Huddinge Sweden

**Keywords:** aerobic exercise, alcohol use disorder, qualitative study, treatment, yoga

## Abstract

**Introduction:**

Exercise is a promising treatment option for individuals with alcohol use disorder, but qualitative studies are lacking. Our aim was to explore experiences of yoga and aerobic exercise among non‐treatment‐seeking adults with alcohol use disorder.

**Methods:**

Semi‐structured qualitative interviews (face‐to‐face or telephone) with 12 participants from a randomised controlled trial. Qualitative content analysis was used to analyse data.

**Results:**

One main category was identified, motivating and maintaining a lifestyle change, including four generic categories: (i) *Initiating factors for lifestyle change*, which describes how the concept of a lifestyle change initiated participants change; (ii) *Influencing lifestyle change*, explains how mood‐enhancing effects from exercise influence exercise behaviours; (iii) *Influencing physical and mental health*, which describes how improvements in physical and mental health influence self‐confidence and self‐esteem; and (iv) *Influencing alcohol consumption,* which describes how exercise reduced alcohol cravings and that success in changing exercise behaviours made participants take healthier decisions regarding their alcohol intake.

**Discussion and Conclusions:**

Exercise may help reduce alcohol intake, especially when presented in the context of a lifestyle change. Being able to self‐select the type of exercise may increase compliance and optimise these benefits. Intentional planning and positive results from exercise may strengthen the individual's self‐efficacy and increase the motivation to change behaviours associated with alcohol consumption.

## 1 INTRODUCTION

Alcohol use disorders (AUD) are common and highly undertreated. Estimates show that less than 20% of those affected receive help for their alcohol‐related problems [1, 2]. Major barriers to treatment are stigma, lack of problem awareness and the desire to self‐manage the problem [3, 4, 5]. In addition to health risks of AUD, those with hazardous alcohol consumption have been found to be less physically active and more sedentary than non‐hazardous drinkers [6, 7], making sedentary individuals with AUD more at risk to non‐communicable diseases such as cardiovascular disease, diabetes [8, 9, 10] and mood disorders [11, 12].

Physical activity is by definition any movement produced by skeletal muscles that requires energy expenditure and includes exercise; a sub‐category of physical activity that is planned, structured and repetitive, with the purpose to improve or maintain physical fitness or health [13]. Yoga is by this definition considered as exercise in this paper, we do however acknowledge that some elements in yoga may not meet this criterion (i.e., breathing techniques, mindfulness). Exercise is known to have positive effects on both physical and mental health, and has been suggested as a promising treatment option for AUD as it is non‐stigmatising, mood‐enhancing and may reduce cravings for alcohol [14, 15] and as a consequence decrease alcohol consumption [16, 17, 18, 19, 20].

Existing research evaluating physical activity interventions for AUD is mainly quantitative focusing on changes in alcohol consumption. A majority of these studies have evaluated aerobic exercise as an adjunct treatment with mixed results. A meta‐analysis, investigating the effects of exercise on AUD, showed no significant reduction in daily alcohol consumption but concluded exercise to be feasible and safe with significant improvements in health outcomes such as depression and physical fitness in the five studies included [19]. Conversely, a more recent meta‐analysis (including seven studies), favoured exercise as an add‐on treatment compared to treatment as usual. Findings showed a significant effect on weekly consumption but not on binge drinking [18].

Qualitative studies describing participants experience of exercise and how it supports, or hinders, in relation to alcohol consumption are lacking. Such information is an important complement to quantitative data and may help to better understand what drives individual behaviour and change during exercise interventions for AUD. Adding to previous research, the aim of this study was to explore experiences of yoga and aerobic exercise among non‐treatment‐seeking adults with AUD.

## 2 METHODS

### 2.1 Design

In this qualitative study, we used the constructivist approach. The constructivist paradigm relies on the assumptions that reality has multiple interpretations, and that the truth is a blend of individuals perception of those realities [21]. This is the reason why we chose to interview our participants as it would have been difficult to explore human experiences as it is lived by using, for example, a questionnaire. Individual semi‐structured interviews were conducted to facilitate participants to describe their experiences of the exercise intervention for AUD (described below). Semi‐structured interviews allow the researcher to prepare questions beforehand, assuring that the interviews stay on topic while allowing interviewees to express their views in their own terms. In comparison to unstructured interviews, semi‐structured interviews provide more comparable qualitative data [22]. When reporting our findings, we adhered to The Standard for Reporting Qualitative Research [23].

### 2.2 Research team and reflexivity

The constructivist paradigm allows various views to be incorporated in the research process [21]. As a result, members of the research group consisted of a registered nurse with a master's degree in nursing (VG), a professor and medical doctor specialising in addiction medicine (SA), an assistant professor specialising in mental health and physical activity (MH) and a registered nurse and professor with long experience of qualitative research (MJ). Both VG and SA have long clinical experience working with AUD treatment. The composition of the research team, in terms of profession and research experience, enabled that various views of the study topic were reflected upon during the different stages throughout the research process. Reflexivity was enabled by continuously questioning the interviewer's background, role and potential effect on the interviewees, both by internal reflection and in dialogue with the research team throughout the whole analysis process.

### 2.3 Sample and setting

**FIGURE 1:**
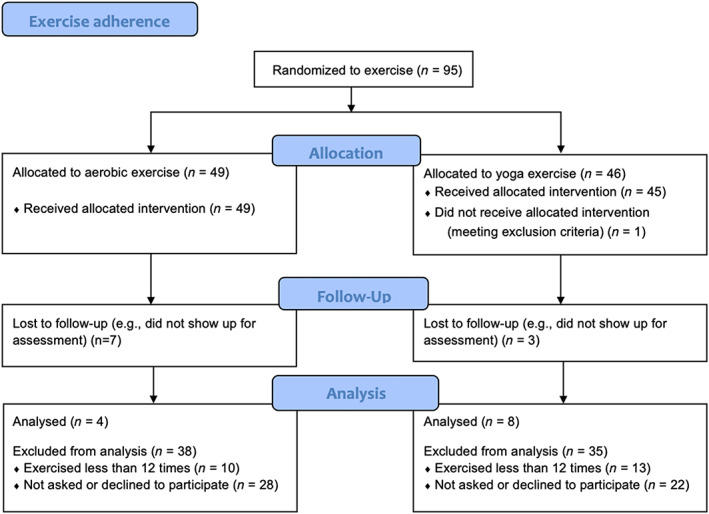
Participant flow chart

Participants were recruited from the FitForChange trial, a randomised controlled trial evaluating the effects of aerobic exercise and yoga as stand‐alone treatments compared to treatment as usual in non‐treatment‐seeking adults with AUD. During a 12‐week period, participants in the exercise arms were asked to exercise at least three times per week and were offered three support sessions (∼30 minutes) with a personal trainer (PT) [24]. Inclusion criteria for this qualitative study were participants randomised to either aerobic exercise or yoga who had exercised a minimum of 12 times during the intervention period and who agreed to be interviewed. The rationale for the inclusion criteria was that the participants needed to have experience of the proposed exercise in order to develop views on the intervention as whole. Participant flow charts are presented in Figure 1.

The aim was to get an even distribution across treatment groups, age and gender. However, due to the COVID‐19 pandemic, recruitment resulted in a sample with a total 12 of respondents, seven women and five men, four from the aerobic exercise group and eight from the yoga group. The aim was not to compare the two different types of exercises included in the intervention, but to explore the participants experiences of an exercise intervention per se. With that in mind, the composition of the sample was considered sufficient to explore experiences of an exercise intervention among non‐treatment seeking adults with AUD. One of the participants had reduced her weekly alcohol consumption from the time of screening to the time of baseline assessment, where the weekly consumption was now lower than Swedish guidelines for women (i.e., ≤9 drinks/week), and one of the participants had previously received alcohol treatment. Interviews were conducted either face‐to‐face at Riddargatan 1, an outpatient clinic located in central Stockholm specialising in AUDs, or by phone and lasted 15–48 minutes (mean 30).

The face‐to‐face interviews were on average longer than the phone interviews. One explanation for this, when assessing the quality of the transcribed interviews, was that face‐to‐face interviews often include discussions not directly relevant to the studied topic (i.e., not included in the analysis). However, both the face‐to‐face and telephone interviews contributed valuable information to the results of the study. All interviews were audio recorded and transcribed verbatim. Demographics of the participants are presented in Table. 1.

**TABLE 1:** Demographic data of participants

ID	Group	Sex	Age	Civil status	Standard drinks per week	DSM‐5 criteria	Number of exercise sessions
1	Yoga	Female	61	Married/co‐habiting	13	5	22
2	Yoga	Male	64	Married/co‐habiting	28	7	22
3	Yoga	Female	33	Married/co‐habiting	18	5	37
4	Aerobic	Male	68	Married/co‐habiting	33	3	34
5	Aerobic	Female	51	Living alone	39	9	36
6	Aerobic	Female	48	Living alone	15	5	21
7	Yoga	Male	62	Living alone	21	3	33
8	Yoga	Male	64	Married/co‐habiting	18	3	44
9	Yoga	Female	70	Married/co‐habiting	9	2	31
10	Aerobic	Female	55	Married/co‐habiting	15	2	33
11	Yoga	Male	63	Married/co‐habiting	17	6	24
12	Yoga	Female	51	Married/co‐habiting	14	5	26

*Note*: DSM‐5, Diagnostic and Statistical Manual of Mental Disorders, fifth edition.

### 2.4 Ethics

The study has been approved by the Regional Ethics Committee (Regionala Etikprovningsnamnden, EPN), Stockholm; DNR: 2017/1380–31. Written informed consent was obtained from all participants in the study.

### 2.5 Data collection and data analysis

Focus areas in the participant interviews were: thoughts on beginning a change; experiences of making changes in alcohol consumption; experiences of physical or mental change during the intervention; and perceptions on what facilitated the intervention and/or made it more challenging. Open‐ended questions were used and follow‐up questions were asked to get richer data. The interview guide, developed by VG, MH and MJ, was initially tested in two interviews by VG. Once they were read and quality approved by MJ (included in the sample), the remaining interviews were conducted by VG at two timepoints, between December 2018 and July 2019 (*n* = 7) and in May 2020 (*n* = 5).

Data were analysed using inductive qualitative content analysis, as described by Elo and Kyngäs [25]. In line with the constructivist paradigm, we used the inductive approach, that is, categories are obtained from the data rather than being determined in advance. The inductive approach is recommended when little is known about the studied topic [21, 25]. As per the analysis of the content, laughs, silence, sighs and so on were omitted from the analysis [25]. To get an understanding of the content as a whole, the transcribed interviews were read once by MJ and several times by VG before starting the process of organising the data. Meaning units, that is, sentences or short sections, were identified if they responded to the aim of the study. As described by Elo and Kyngäs [25] an open coding of the data was performed, that is, headings and notes (codes) that described the meaning units were written down. Subsequently, codes with similar labels were combined to generate sub‐categories which resulted in 43 codes and 11 sub‐categories. Sub‐categories were grouped into generic categories that thereafter were grouped into one main category (Table 2). The process of combining generic categories into main categories is called abstraction and can continue as far as the researcher finds reasonable and possible [25]. During the analysis process, there was a continuous discussion between VG and MJ, to ensure the credibility and trustworthiness of the analysis. An example of the analysis process is presented in Table 2. The analysis process and the result were discussed with SA and MH, who provided constructive feedback and clarifying questions throughout the process which ensured that the results described the meanings of the contents of the generic categories. Since the interviews were made in Swedish, the analysis was conducted in Swedish to not alter, distort or overinterpret the meaning of the participants' descriptions. In the process of writing the result, it was written in English from the beginning and the illustrative quotes were translated from Swedish to English. One of the members in the research team (MH) has English as native language which reduced to risk of misinterpretation during the process.

**TABLE 2:** Example of the analytical process

Meaning unit	Code	Sub‐category	Generic category	Main category
*‘I had seen an ad in the newspaper, maybe a month or a couple of months before with the message; do you drink too much and exercise too little? And I thought, that fits me'*.	Appealing	Factors influencing start of treatment	Initiating factors for life‐style change	Motivating and maintaining a lifestyle change
*‘Yes, first of all it was a whole new world that I have never been to, just going to the gym is new to me, and then ending up in a yoga studio with 20, 65 plus women as the only man, felt very strange'*.	Uncomfortable	Factors influencing exercise	Influencing lifestyle change	
*‘It was of great importance, partly that I feel better, calmer, yes more satisfied and, yes it did a lot for my health to start exercising'*.	Emotion	Mental well‐being	Influencing physical and mental health	
*‘Partly when you are happy and satisfied after exercising, the interest in getting a reward by drinking decreases'*.	Reward	Factors influencing the consumption	Influencing alcohol consumption	

## 3 RESULTS

The analysis identified one main category, motivating and maintaining a lifestyle change, with four generic categories: (i) Initiating factors for lifestyle change; (ii) Influencing lifestyle change; (iii) Influencing physical and mental health; and (iv) Influencing alcohol consumption.

### 3.1 Initiating factors for lifestyle change

What appealed to the participants and made them apply to the study was that the intervention focused on increased exercise, rather than giving directions on how to reduce or stop drinking. Being both physically inactive and drinking too much alcohol, participants instinctively felt they were suitable for the intervention. All but one of the participants emphasised that they would never seek specialist treatment as they did not see themselves as having alcohol problems requiring professional help. However, undergoing a lifestyle change was a suitable approach for their excessive alcohol consumption. One participant mentioned previous experience of drinking less when being physically active and all of them have had the wish to be randomised to one of the exercise groups.

*‘And this, the advertisement was just, it felt so nice that it was: Do you want to change your behaviour, change your pattern? Then it became different in a way than if it would have said: Do you want to quit? Then I probably wouldn't have applied, I don't think so'*. (1)

### 3.2 Influencing lifestyle change

Participants described the importance of engaging in a suitable type of exercise. When referring to exercise as suitable, it was the type that overall gave a positive feeling. This involved doing a type of exercise that was fun, gave a feeling of relatedness and that the exercise was at an appropriate level. However, this was also very individual and affected the type of exercise classes performed. Many participants experienced group exercise and the feeling of fellowship with others as fun and motivating.

*‘And I thought it was great fun, this kind of exercise when you are together, I think group exercise is very fun'*. (10)

When participants found a group where everyone was at the same level regarding previous exercise experience, it resulted in a feeling of relatedness which influenced exercise behaviours in a positive way. However, being in a group could also make you feel left out, with individuals in better shape, different age or gender which affected the experience negatively. This raised a feeling of insecurity and exercising became uncomfortable. One participant emphasised that the feeling of fellowship with others was ruined by an uneven gender distribution in the exercise class and found the environment unwelcoming.

Most participants wanted to do other types of exercise than those available during the trial and expressed a preference to alternate between yoga and aerobic exercise, while others wanted to perform strength training as well. Not being able to vary the training was considered limiting, having a negative effect on exercise habits. Some of the participants were also disappointed by being randomised to an exercise group not of their choice which influenced their motivation to exercise negatively.

*‘What I missed, I wasn't supposed to do strength training or practice yoga and such and I missed that a bit. … So, it was probably the lack of not being able to vary the exercise … It would have felt a little more fun. Some classes I went on were very boring. A bit more fun, and then I think I might have managed to do some more exercise sessions a week'*. (5)

Exercising at the appropriate level meant exercising at an intensity according to one's own ability, especially when previously being physically inactive. Participants appreciated having the option to choose different types of exercise (within the randomised group), as it facilitated finding an exercise group at an appropriate intensity. Some participants emphasised that they had become less flexible and immobile with age, making yoga an appropriate type of exercise. When the intensity level was too high and did not correspond to their own fitness level, it was perceived as difficult and could negatively affect the exercise experience. However, it was also important that the exercise was not perceived as too easy. Balancing the right level and intensity so that the exercise became sufficiently strenuous was mood‐enhancing and had a positive influence on exercise behaviours.

*‘When it is strenuous enough and I get this positive feeling in the body, something happens in the body that, that simply makes me feel good'*. (7)

Most participants mentioned that the biggest obstacles to exercise were finding the motivation and time to go to the gym. Planning the weekly exercise in advance, having close access to fitness centres and a wide range of classes made it easier to maintain exercise behaviours. Online training was thought of as helpful when it was impossible to find time to go to the gym. However, some participants perceived it as boring demanding a higher level of self‐discipline than exercising at a gym.

*‘But then you could do the online training as well, it kind of helped that you could actually do your own exercise. So, it was quite liberal anyway, that you could throw in a video once or twice if you realised that you were not able to do three*'. (8)

Several participants emphasised the monitoring of progress as a key factor for maintaining motivation to exercise. For some, being part of a research trial and loyal to the cause of research was described as motivating. Self‐monitoring, by using an exercise calendar, was also helpful. Being contacted by a PT, who introduced them to the gym, made it easier to start exercising. The PT was expected to be humble and provide guidance adapted to participants experience, ability and goals. However, in addition to only receive motivating sessions, most participants expressed a desire of having individual training with the PT. The instructor holding the exercise class was important for both maintaining motivation during class and to maintain exercise behaviours. Clear instructions, competence and the ability to motivate people were important qualities for an instructor and made participants push themselves a bit further.

*‘Yes, exactly, when there were good instructions, and you got several alternatives to a position and when they did not assume that everyone is the same … and humility and some kind of normal nice, positive attitude, then it was nice, then it was good*'. (3)

Participants mentioned that the healthy lifestyle approach enabled them to talk to family and friends about their excess alcohol intake, as it was perceived as less stigmatising to say that they were doing a lifestyle change rather than alcohol treatment. Some participants said they would recommend exercise as treatment for AUD, and most participants mentioned having continued to exercise after the trial in order to maintain their lifestyle change.

### 3.3 Influencing physical and mental health

Both aerobic exercise and yoga improved participants physical strength and energy, and some mentioned a reduction in body size and weight. One participant experienced pain relief after starting to exercise (aerobic exercise), improving the persons quality of life. Overall, these physical improvements contributed to continue exercising, and also led to increased everyday physical activity (i.e., walks). Participants doing aerobic exercise experienced how quickly their physical fitness and stamina improved.

*‘It was important because I saw how quickly my fitness improved, I felt that when I went on a cycle session in the beginning I almost died, and after a while it wasn't the same, it wasn't as hard, I managed to do more. I managed more resistance, so I quickly noticed how fitness and strength improved*'. (6)

Yoga was experienced as a calm, yet physically demanding type of exercise and the yoga postures were initially experienced as challenging for some. With time and practice the participants improved their balance and became more agile.

*‘You realised your limitations when it came to stretching and balance, but that had to do with that the first few times it was quite awkward with some of these more strenuous exercises in terms of balance. But even so, after 10 times you became a little better, that was it*'. (8)

Exercise also influenced the mental well‐being. Most participants experienced an increased mental energy making them alert and more productive. One participant described becoming more creative, as the best ideas came while exercising. Paradoxically, participants also felt that they became calm and more harmonic, which affected their everyday life in a positive way. One participant described becoming less anxious after having started to exercise. Breathing exercises during yoga were experienced as both calming and regenerating and two participants experienced how their mind, body and soul became one while practicing yoga. Positive mood states, such as feeling happy, generated a more positive outlook on life and changed every day behaviours.

*‘Yes, I probably feel sort of, yes happier, calmer, and happier … maybe a little more social, socially open towards my environment and, I used to feel very abject and, yes I'd lost my spark and hope a little before I got started. So, I feel more positive, a little more open, generally a bit happier, I believe'*. (5)

After completing an exercise session, the participants felt proud of themselves and emphasised how they started to value their body and health more. Exercising gave participants hope that change is possible, which strengthened the belief of being capable of change. Participants began to believe in themselves, their self‐confidence and self‐esteem grew.

*‘Because I feel more satisfied with myself. I don't walk around with the anxiety that I had before. I felt pretty bad as a human many times, actually. I swept it under the carpet all the time, but I didn't feel well, and I felt useless for not doing something about it. That I didn't deal with it. So, this makes me feel, you get better self‐esteem and … I think I have, yes … Yes, I actually have better self‐esteem, and feel that I am capable, I'm pretty good quite simply, I think'*. (1)

Although two of the participants did not experience any change in sleep behaviour, well‐being was connected to improved sleep. Some participants described that they fell asleep more easily, woke up feeling rested and some experienced having a deeper sleep. Better sleeping habits influenced both their physical and mental health.

*‘Yes, among other things, I used to have a very hard time falling asleep, and then I could fall asleep very late, and then I had to get up very early and it just continued like that. I have no problems at all with sleep, it is completely gone, I fall asleep, I sleep like a log, I wake up and I am alert. I mean, well I couldn't explain it better'*. (3)

However, some participants were unsure if it was the exercise that gave them a good sleep or if it was the combination of exercising more and drinking less alcohol.

### 3.4 Influencing alcohol consumption

Most participants reduced their alcohol consumption, both in number of drinks per occasion and number of days drinking per week, resulting in a consumption below the Swedish recommended guidelines. This was partly a result of reduced alcohol craving, especially on days having exercised. Although this feeling was distinct for most participants, two individuals described that their alcohol consumption remained unchanged despite reduced urge for alcohol.

*‘Yes, but I actually think that after exercise, the craving decreases. But I have been drinking anyway, that's a different thing, but it hasn't been quite the same craving*'. (5)

The reduction in alcohol consumption was also attributed to feeling strong, fresh and healthy after exercise, which participants did not want to destroy by drinking alcohol. This newfound desire to value their own health, influenced alcohol habits among participants and several participants described alcohol as something contaminating the body.

*‘I felt, I don't know what it means, it felt like, like I soiled my system, my internal body system with things that shouldn't be there, and it became very clear when I exercised'*. (3)

Exercise was viewed as the polar opposite to alcohol intake, where exercise stood for the positive and alcohol the negative. The participants emphasised how they felt proud after exercising, having done something positive for their own health. Exercise itself was perceived as rewarding and reduced the need to reward themselves with alcohol.

*‘I'm not a real behaviorist but doing yoga after work was a reward in itself and it created a well‐being and therefore the need to find a new reward disappeared when you came home'*. (11)

Scheduling the exercise did not only help to maintain exercise habits but was also used as a behavioural strategy to reduce alcohol consumption. By scheduling the exercise in the morning, many participants chose to drink less or not at all the night before. Some also scheduled their exercise after work or late in the evening.

*‘Knowing that I had planned to exercise, could also lead to not going with friends if they were going for a drink after work or something, the fact that I had decided to exercise also meant that I reduced my alcohol intake because there was a plan'*. (6)

## 4 DISCUSSION

This is the first study exploring experiences of exercise among non‐treatment‐seeking adults with AUD. Our study found that the concept of making a lifestyle change was attractive for participants and initiated the willingness to change their alcohol consumption. Being able to engage in a self‐selected type of exercise was mood‐enhancing and having someone monitoring progress was of importance to maintain motivation. Results also showed that exercise improved participants physical‐ and mental health as well as their self‐confidence and self‐esteem. The decrease in alcohol consumption was related to changes in alcohol cravings and success in changing exercise habits increased motivation to change alcohol consumption.

Findings related to what influenced participants to initiate change support previous research suggesting that individuals with AUD are interested in exercise‐based treatments and willing to engage in them. This has been reported in both clinical settings [26, 27] and in non‐treatment seeking individuals [24, 28]. All participants found the exercise interventions suitable and appealing, as they focused on a healthy lifestyle rather than solely targeting consumption and alcohol‐related problems. This is a highly significant finding considering the large treatment gap between those affected by AUD and those in treatment, suggesting that exercise should be included by health providers in their lists of interventions for individuals with AUD. The healthy lifestyle approach is also in line with real‐world scenarios where individuals changing their alcohol consumption without treatment have reported simultaneous health behaviour changes, including undertaking new exercise habits [29]. This highlights the benefits of multiple health behaviour changes (MHBC), that is, interventions that promote two or more health behaviours simultaneously [30].

A theoretical model applicable on multiple health behaviour change is the Compensatory Carry‐Over Action Model (CCAM) [31]. The model suggests that the intentional planning and positive results from one behaviour can strengthen self‐efficacy and spill over to other behaviours, giving the ability to handle tempting situations leading to unhealthy behaviours. The model relies on five assumptions. First, different health behaviours interrelate and just as unhealthy behaviours tend to cluster, healthy ones do as well. Second, health behaviours stem from emotionally higher‐level goals. In this study, the participants had clustering unhealthy behaviours (physically inactive and drinking too much) and the higher‐level goal of becoming healthier.

One important factor that influenced lifestyle change was the ability to engage in exercise of their own choice. The choice of exercise type was highly dependent on individual preferences but had the common denominator of being mood‐enhancing; a finding consistent with previous research showing positive changes in mood states in response to acute exercise [14, 32]. This resonates with the CCAM's third assumption, that new behaviours need to be intended and planned, with the key components individualisation and personalisation [31]. This was described by participants as necessary to prevent falling into old bad habits. Other factors influencing participants lifestyle change was the need of having someone monitoring their progress, and that exercising with individuals in better shape can be a negative experience. Both these findings are supported in a qualitative study by Sari and colleagues exploring barriers to exercise in individuals with AUD [33].

Most participants expressed an increase in self‐confidence and felt more capable of change as they started to exercise. The concept of self‐efficacy, the belief that one can successfully perform a given activity, is an important factor of the third assumption in the CCAM model. The assumption holds that positive results of one behaviour (exercise) can affect the motivation and willingness to achieve change in another (alcohol consumption), with self‐efficacy moderating the relationship between the two behaviours [31].

Regarding factors influencing physical and mental health, findings showed that participants in both exercise groups improved their physical and mental strength and energy, and they became less stressed, calmer and happier. This is consistent with health benefits of exercise reported previously [34]. However, two participants specifically mentioned experiencing a spiritual connection while practicing yoga, referring to the breathing exercises. Although spirituality is a broad concept requiring a deeper understanding of its mechanisms, yoga‐based exercise is sometimes associated with mindfulness and spirituality [35].

Factors influencing alcohol consumption support previous research suggesting that exercise decreases alcohol craving through changes in negative reinforcements (i.e., drinking to reduce tension or worries) [14]. In addition, the desire to drink alcohol as a reward was inhibited as a consequence of mood‐enhancing effects and the feeling of pride after having exercised. Many participants minimised the risk of alcohol intake by planning and scheduling their exercise. One interesting dimension of the reduction in consumption was participants experience of valuing their health higher as they started to exercise. Alcohol was perceived as something that would ruin their efforts and contaminate the body. These behaviour‐specific processes (i.e., intentions, plans and experiences) are described in the fourth assumption of the CCAM model, known as the carry‐over mechanism. This means that processes from one behaviour (exercise) will interrelate, spill over and affect the second behaviour (alcohol consumption). The carry over‐mechanism also holds that by doing one behaviour you can compensate for not doing the another [31]. However, this type of compensatory behaviour was not described by the participants in our study. Consequently, by prioritising higher‐level goals and changing multiple behaviours in line with a healthy lifestyle, participants reduced tempting situations at the same time as their well‐being increased, which is in line with the fifth assumption in the CCAM model [31].

## 5 LIMITATIONS

The choice to include participants who had exercised at least 12 times during the intervention may have excluded those who found it challenging to start the exercise regimen. However, including people with very little or no experience of the exercise intervention could have resulted in experiences solely related to external factors (such as no time or lack of motivation). Due to the small sample of 12 participants, it can be argued that a larger sample could have given richer data. However, with 12 interviews the information power was sufficiently large in regard to aim, sample specificity, quality of dialogue and analysis method [36]. Participants were part of the FitForChange trial and the result of a convenience sampling (i.e., participants in the trial who accepted to be interviewed), which may have influenced the results. In regard to the sampling, more individuals from the yoga group were interviewed, but the distribution between sex and age does not differ greatly from the randomised controlled trial. By exemplifying the analysis process and illustrating results using quotations, the liaison between collected data and results is visualised for the reader and will contribute to the transparency and credibility of the study. [25].

## 6 CONCLUSION

Given that the vast majority of those with AUD do not seek treatment because of stigma and lack of problem awareness, changing alcohol habits may be easier to accomplish in the context of a lifestyle change with a focus on increased exercise. To motivate and maintain these changes, one needs the capacity to choose where, when and how exercise is performed. Participation in regular exercise can have positive mood‐enhancing effects resulting in lower alcohol consumption. These behaviour‐specific processes can be better understood by using the CCAM. In the context of increasing treatment seeking among individuals with AUD, a lifestyle change may attract those reluctant undergoing usual care. By using physical activity on prescription, exercise can for example be implemented in health care and used as a gateway to access alcohol treatment.

Future qualitative research should include a theory‐based evaluation of how behaviours associated with exercise and alcohol consumption change and influence each other, and also include those with little or no experience of the exercise intervention to possibly explore other challenging aspects of exercise as a possible lifestyle change in treatment for individuals with AUD. Also, randomised controlled trials with long‐term follow‐ups are needed to examine changes over time.

## AUTHOR CONTRIBUTIONS

Victoria Gunillasdotter: Conceptualisation; data curation; formal analysis (lead); contributed to funding acquisition; investigation; methodology; project administration; validation; visualisation; writing original draft. Sven Andréasson: Conceptualisation; funding acquisition; resources; supervision; review and editing. Mats Hallgren: Conceptualisation; funding acquisition; supervision; review and editing. Maria Jirwe: Conceptualisation; formal analysis (supporting); methodology; supervision; review and editing.

## FUNDING INFORMATION

This work was supported by the Swedish Research Council for Health, Working Life and Welfare (FORTE) (grant number: 2016‐07113), and the Ekhaga Foundation (grant number: 2018‐0042). Funders had no role in the study design, the collection, analyses, and interpretation of data, writing of the report, or in the decision to submit the article for publication.

## CONFLICT OF INTEREST

The authors declare no conflict of interest.
